# Acute neuromuscular, metabolic, and perceptual responses to low-load isokinetic exercise under varying percentages of arterial occlusion pressure

**DOI:** 10.3389/fphys.2026.1785040

**Published:** 2026-03-04

**Authors:** Junwei Xia, Ziyuan Yuan, Lili Cong, Yuan Chen, Tianqi Zhao

**Affiliations:** 1 Department of Leisure and Sports, Kangwon National University, Samcheok, Republic of Korea; 2 Department of Economics and Finance, Kangwon National University, Samcheok, Republic of Korea; 3 Early Childhood Education, Kangwon National University, Samcheok, Republic of Korea; 4 School of Information and Intelligent Manufacturing, Fuyang Normal University, Fuyang, Anhui, China; 5 Department of Leisure and Sports, Physical Education Measurement and Evaluation Lab, Kangwon National University, Samcheok, Republic of Korea

**Keywords:** arterial occlusion pressure, blood lactate, isokinetic dynamometry, perceived exertion, surfaceelectromyography

## Abstract

**Objective:**

To examine whether graded arterial occlusion pressure (AOP) during low-load isokinetic exercise differentially modulates neuromuscular activation, metabolic stress, and perceptual strain, and to identify an occlusion level that maximizes stimulus while minimizing perceived burden.

**Methods:**

Twelve healthy young men (21.3 ± 1.6 years) completed four randomized, counterbalanced sessions under 0%, 60%, 70%, and 80% AOP. During each session, isokinetic knee extension/flexion was performed at a low load under the assigned occlusion condition. Muscle activation was quantified using surface EMG and expressed as %EMGmax. Capillary blood lactate (BLa) was assessed at baseline and during recovery, and rating of perceived exertion (RPE) was recorded immediately after exercise.

**Results:**

Occlusion pressure produced a clear dose–response pattern in metabolic stress and neuromuscular demand. Both %EMGmax and BLa increased with higher AOP, with the most consistent elevations occurring at ≥70% AOP (p < 0.05). Importantly, raising occlusion from 70% to 80% AOP did not provide additional gains in %EMGmax, whereas RPE increased significantly at 80% AOP compared with 70% AOP (p < 0.05).

**Conclusion:**

Low-load isokinetic exercise performed at 70% AOP elicits robust neuromuscular and metabolic stimulation without additional gains relative to 80% AOP while imposing a substantially lower perceptual load. These findings support 70% AOP as a practical “compromise point” for acute BFRT prescription when balancing training stimulus and tolerability.

## 1 Introduction

Currently, research directly comparing acute neuromuscular and perceptual responses during blood flow restriction training (BFRT) at different percentages of arterial occlusion pressure (AOP) (e.g., 60%, 70%, and 80% AOP) in healthy adults remains relatively limited. This evidence gap constrains our understanding of how occlusion pressure levels modulate the intensity of training stimuli, the magnitude of metabolic responses, and exercise tolerability, which in turn leaves evidence-based pressure prescription recommendations insufficiently defined.

Recent evidence has begun to directly compare acute neuromuscular, metabolic, and perceptual responses across 0%–80% AOP during traditional low-load resistance tasks (e.g., barbell back squats) (Zhu et al., 2025). However, it remains unclear whether the apparent diminishing-return pattern at ≥70% AOP generalizes to an open-chain isokinetic model, where angular velocity is constrained and torque is regulated across the full range of motion.

BFRT is a low-load, short-duration resistance training modality that utilizes an inflatable cuff applied to the proximal limb to partially restrict arterial inflow and limit venous return. Within this artificially induced hypoxic and metabolic accumulation environment, the body can achieve adaptive stimuli similar to traditional high-load training at significantly lower loads; the underlying mechanisms facilitating muscle hypertrophy and strength gains have been synthesized in several systematic reviews (Centner et al., 2019; Lixandrao et al., 2021; Slysz et al., 2016). As a core technique derived from Japanese “KAATSU” training, BFRT is widely applied in athletic performance optimization, sports rehabilitation, and the prevention of clinical muscle atrophy (Moriggi et al., 2021; Nakajima et al., 2020; Abe et al., 2010).

Extensive research indicates that under isokinetic conditions (e.g., using a Biodex dynamometer), low-intensity BFRT set relative to peak torque (PT) (typically ∼30%–40% PT) can induce significant strength gains over several weeks. However, long-term evidence regarding muscle hypertrophy and volume within isokinetic training systems remains relatively inconsistent and warrants further investigation (Biral et al., 2025; Hill et al., 2018; Sakuraba and Ishikawa, 2009; Welsh et al., 2024). These training effects are generally attributed to elevated metabolic stress under restricted blood flow and changes in motor unit recruitment patterns driven by fatigue (Fahs et al., 2015; Scott and Loenneke, 2022). Consequently, within the BFRT prescription framework, cuff pressure serves as a critical variable determining the magnitude of metabolic stimulus, subjective discomfort, and training sustainability (Mouser and Mattocks, 2017).

Theoretically, BFRT cuff pressure should achieve sufficient venous restriction without completely occluding arterial perfusion to maximize metabolic stress while ensuring safety (Loenneke et al., 2014). However, the pressure required to reach a similar degree of blood flow restriction is significantly influenced by factors such as cuff width, limb circumference, subcutaneous fat thickness, and individual vascular health (Buckner et al., 2020). Excessively high pressures may trigger significant pain, intolerance, or potential adverse events, while insufficient pressure may fail to generate an effective metabolic stimulus, thereby weakening the training effect (Günter and Behringer, 2023; Mouser and Mattocks, 2017; Nascimento et al., 2022). Furthermore, using a uniform absolute pressure (e.g., fixed at 180–220 mmHg) fails to produce consistent restriction levels across individuals, leading to a “bidirectional bias”—under-occlusion in larger limbs and over-occlusion in smaller limbs (Cook et al., 2014; McEwen et al., 2019; Patterson et al., 2019).

For these reasons, current international consensus and guidelines generally recommend using a percentage of individualized AOP as the standard for pressure prescription (Counts et al., 2016; Patterson et al., 2019; Patterson and Hughes, 2021). Determining AOP via Doppler ultrasound or PPG and setting training pressure accordingly enhances the reliability and inter-individual comparability of the intervention (Hughes et al., 2018; Smilios et al., 2022). Nevertheless, the “optimal range” for AOP percentage remains a subject of debate; some studies suggest that moderate ranges (e.g., 50%–80% AOP) may better balance efficacy and tolerability, yet the optimal pressure for different populations and exercise structures remains unclear (Das and Paton, 2022; Roehl et al., 2023). Notably, a substantial proportion of existing BFRT literature fails to provide sufficient justification for the selected AOP percentages, which compromises the reproducibility of results and limits the generalizability of prescriptions (Clarkson et al., 2020).

Clarifying the effects of different relative pressure levels on acute neuromuscular responses and perceptual load is of significant theoretical and practical importance. Therefore, this study utilizes Doppler ultrasound to precisely determine individual AOP and systematically compares the acute effects of low-load BFRT at 60%, 70%, and 80% AOP in healthy young men. Specifically, this study evaluates muscle activation (%EMGmax), blood lactate concentration, and Rating of Perceived Exertion (RPE) to provide evidence-based recommendations for practical pressure prescription.

The experimental hypotheses are as follows:

Null Hypothesis (H_0): There are no significant differences in muscle activation, blood lactate concentration, or RPE across different AOP levels during BFRT. Alternative Hypothesis (H_1): Compared to 60% AOP and control conditions, 70% and 80% AOP will elicit higher muscle activation, greater lactate accumulation, and increased subjective fatigue.

## 2 Materials and methods

### 2.1 Participants

An *a priori* power analysis was conducted using G*Power 3.1.9.7 software to estimate the sample size for a repeated-measures analysis of variance (RM-ANOVA, within-subject design) (Faul et al., 2007). With an alpha level of 0.05, a power (1-β) of 0.80, and an expected medium effect size (partial ηp^2^ = 0.06, corresponding to Cohen’s f ≥ 0.25), the minimum required sample size was determined to be 12 participants. Consequently, 12 healthy male university students (age 21.3 ± 1.6 years) were recruited for this study.

All participants provided written informed consent before the commencement of the study. The study protocol was approved by the Institutional Review Board (IRB) of Kangwon National University (IRB No.: KWNUIRB-2025-07-002-001). Inclusion criteria required participants to be healthy males aged 18–30 years with no known cardiovascular, metabolic, or neuromuscular diseases, and the ability to perform low-load resistance training. Exclusion criteria included a history of diagnosed thrombosis, abnormal electrocardiogram results after exercise, resting blood pressure exceeding 160/100 mmHg, and a difference in bilateral thigh circumference greater than 0.5 cm.

### 2.2 Experimental design and procedures

The study employed a randomized crossover, repeated-measures design. To eliminate order effects, participants were randomly assigned to one of four pressure sequences based on a balanced Latin square (Williams design). The procedure consisted of a familiarization session (Visit 0) and four formal experimental visits (Visits 1–4), with at least 7 days between sessions. All visits were conducted at the same time of day for each participant.

#### 2.2.1 Arterial occlusion pressure (AOP) measurement

AOP was determined using Doppler ultrasound under standardized conditions. It should be noted that AOP is posture-dependent; therefore, although measurement procedures were consistent across sessions, the effective occlusive stimulus during the seated isokinetic exercise may have differed slightly due to hydrostatic influences.

AOP was measured in a quiet environment using a color Doppler ultrasound system (WISONIC Piloter S, China) at the proximal end of the participant’s thigh. A 50-mm wide pneumatic cuff was used, and 100% AOP was defined as the cuff pressure at which arterial blood flow in the femoral artery disappeared, as assessed by Doppler ultrasound. This method has been validated for its clinical reliability (Masri et al., 2016). All measurements were performed by a professional with over 3 years of experience in vascular ultrasound, and the intra-class correlation coefficient (ICC) between two independent measurements was 0.993. The average AOP of the participants was 241.30 ± 21.50 mmHg. Body position was standardized and kept constant across all AOP determinations; nevertheless, AOP is posture-dependent, and any mismatch between the measurement position and the seated isokinetic task could introduce systematic deviation in the effective occlusive stimulus.

Because arterial occlusion pressure is posture-dependent, the reported %AOP values should be interpreted as standardized reference pressures rather than the exact in-task occlusive stimulus during the seated isokinetic exercise.

#### 2.2.2 Exercise protocol (biodex isokinetic task)

During each formal visit, participants completed lower-limb knee extension/flexion training on a Biodex isokinetic system. The exercise intensity was set to 30% of the maximum peak torque (PT) measured during the screening visit. The training followed the classic BFRT protocol: 4 sets with a repetition scheme of 30 + 15+15 + 15, and 60-s rest intervals between sets (Patterson et al., 2019). Key settings included an angular velocity of 60°/s, full pain-free ROM, concentric/eccentric mode, and gravity correction. Cuff pressure was inflated before the first set and maintained during rest intervals until the completion of the final set. The isokinetic exercise protocol and key procedures are summarized in Table 1.

**TABLE 1:** Biodex isokinetic exercise protocol under different AOP conditions.

Procedures	Visits	Pressure condition (%AOP; individualized mmHg)	Exercise/Measurement parameters	Duration (min)
Familiarization (visit 0)	Visit 0	0% AOP or low pressure (optional; not analyzed)	Practice biodex setup and range of motion; cuff placement & inflation familiarization; RPE instruction; MVC practice	20–30
Warm-up	Visits 1–4	–	Dynamic stretching (lower limbs)	10
Main exercise (biodex)	Visits 1–4	One of: 0%, 60%, 70%, 80% AOP (order randomized & counterbalanced) (0% AOP: Cuff worn but not inflated)	Isokinetic knee extension/flexion; angular velocity 60°/s; ROM: Full, pain-free ROM (individualized, consistent across visits, and recorded); concentric/eccentric mode; gravity correction applied; target torque ≥30% pre-test PT with real-time biofeedback; standardized seat/axis/straps	15–25
BFR application (BFR sessions only)	Visits 1–4 (BFR)	60%/70%/80% AOP	Pneumatic cuff at proximal thigh; target pressure = (%AOP) × individual AOP (mmHg); maintain pressure during each set; monitor symptoms; if intolerable, reduce by 10 mmHg steps and record	Included
Outcome measurements	Visits 1–4	–	sEMG recorded throughout exercise; blood lactate at pre, post-0, post-5, post-10; venous blood at pre and post-5 (5 min after exercise); RPE recorded after each session	Included
Cool-down	Visits 1–4	–	Slow walking + stretching	10

Target pressure (mmHg) = (% AOP) × individual AOP (mmHg); 0% AOP, served as the no-BFR, control. If excessive discomfort occurred, cuff pressure could be reduced in 10 mmHg steps and recorded.

### 2.3 Data collection and processing

#### 2.3.1 Surface electromyography (sEMG)

**FIGURE 1:**
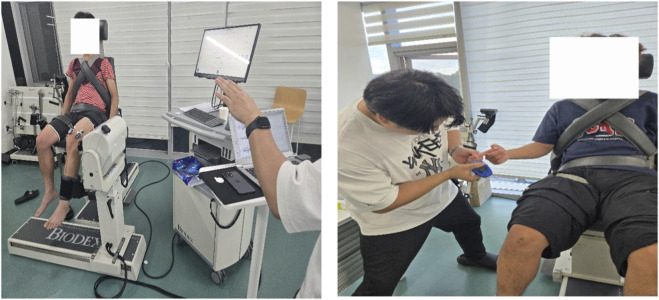
Schematic of surface electromyography (sEMG) and blood lactate (BLa) collection.

sEMG signals were collected following the SENIAM guidelines (Hermens et al., 2000). The BTS FREEEMG 1000 system (sampling rate 1,000 Hz) was used to monitor the rectus femoris (RF), vastus medialis (VM), vastus lateralis (VL), biceps femoris (BF), semitendinosus (ST), and gluteus maximus (GM). Raw signals were processed with a fourth-order Butterworth band-pass filter (20–450 Hz), full-wave rectified, and converted to root mean square (RMS) amplitude using a 50-m moving window. EMG amplitude outcomes are reported as %EMGmax and were normalized to the maximal EMG amplitude observed during maximal voluntary contraction (MVC) trials for each muscle (Konrad, 2005). For each muscle, three MVC trials were performed and the highest EMGmax value was used for normalization. Task-related %EMGmax was calculated as the mean RMS across all repetitions performed in the four sets, averaged across the concentric phase only. The measurement setup is illustrated in Figure 1. MVCs were performed at each visit (i.e., for each AOP condition). EMGmax was defined as the peak RMS EMG (50-m moving window) during MVC trials (three trials; maximum retained), and MVCMAX was defined as the highest peak torque across MVC trials for knee extension and flexion at the same visit.

MVC peak torque (MVCMAX) was recorded concurrently during MVC testing on the Biodex dynamometer. Participants performed maximal isometric knee extension and knee flexion efforts at a fixed joint angle (standardized within participant across visits), with 60 s rest between trials. The highest peak torque value was taken as MVCMAX for each action. Descriptive MVCMAX and EMGmax values used for EMG normalization are provided in Supplementary Table S1.

#### 2.3.2 Blood indicators

Blood Lactate (BLa): Measured via fingertip blood samples at baseline (Pre), immediately after exercise (Post-0), and at 5 (Post-5) and 10 min (Post-10) of recovery.

**FIGURE 2:**
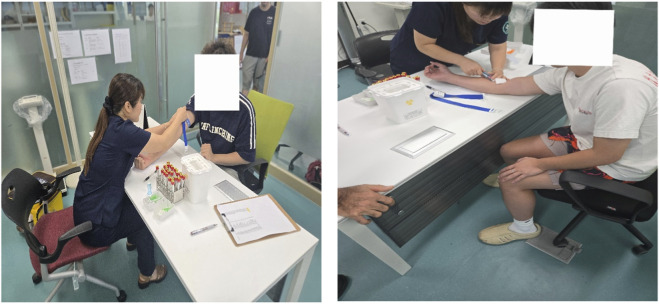
Venous blood collection.

Biochemical and Endocrine Markers:Venous blood (2 mL) was collected from the antecubital vein before exercise and at a standardized time point 5 min after exercise (Post-5) to capture acute hormonal and inflammatory responses (Kraemer and Ratamess, 2005; Pedersen and Febbraio, 2008). Samples were centrifuged (1,500 × g, 10 min) and stored at −80 °C. Interleukin-6 (IL-6) and testosterone (TST) were analyzed using the Roche cobas platform (c702 and e801 modules). Venous blood collection procedures are shown in Figure 2.

#### 2.3.3 Rating of perceived exertion (RPE)

In each pressure condition, participants assessed their perceived exertion immediately after the fourth set using the Borg 6–20 scale (Borg, 1998).

### 2.4 Statistical analysis

Data are presented as mean ± standard deviation (SD). Normality was assessed using the Shapiro-Wilk test, and sphericity was tested using Mauchly’s test (with Greenhouse-Geisser correction applied where necessary). A one-way RM-ANOVA was used for %EMGmax and RPE, while a two-way (Time × Pressure) RM-ANOVA was used for BLa and blood markers. Significant differences were further analyzed using Bonferroni *post hoc* tests. The significance level was set at p < 0.05, and effect sizes were reported as partial eta squared ηp^2^.

## 3 Results

### 3.1 Comparison of muscle activation levels (%EMGmax) across pressure conditions

The repeated-measures ANOVA revealed a significant main effect of occlusion pressure on the activation levels of all analyzed muscles (all p < 0.001, ηp^2^ = 0.58–0.76). Overall, %EMGmax increased with rising pressure, reaching peak levels at 70% AOP (52.57%–54.57% EMGmax). Neuromuscular activation outcomes across conditions are summarized in Table 2.

**TABLE 2:** Lower-limb muscle %EMGmax under different AOP conditions with RM-ANOVA main-effect statistics (Mean ± SD, n = 12).

Muscle	0% AOP	60% AOP	70% AOP	80% AOP	F (3,33)	p-value	Partial ηp^2^	95% CI for ηp^2^
Rectus femoris (RF)	44.67 ± 2.62	47.23 ± 2.62	52.57 ± 3.07***†	49.51 ± 2.25*	18.15	<0.001	0.62	[0.38, 0.75]
Vastus lateralis (VL)	44.86 ± 2.15	47.53 ± 1.91*	52.94 ± 2.77***†	49.76 ± 2.92**	26.89	<0.001	0.69	[0.51, 0.81]
Vastus medialis (VM)	45.97 ± 2.68	48.85 ± 2.05	54.28 ± 2.20***†	51.31 ± 2.84**	29.48	<0.001	0.73	[0.54, 0.82]
Biceps femoris (BF)	46.23 ± 1.75	48.78 ± 1.95*	54.57 ± 2.38***†	51.29 ± 2.71***	34.53	<0.001	0.76	[0.59, 0.84]
Semitendinosus (ST)	44.78 ± 2.00	47.46 ± 2.69**	52.86 ± 3.49***†	49.59 ± 3.21**	19.8	<0.001	0.62	[0.41, 0.76]
Gluteus maximus (GM)	44.94 ± 1.49	47.60 ± 2.64	53.09 ± 2.96***†	49.76 ± 2.70**	26.55	<0.001	0.71	[0.51, 0.81]

AOP, arterial occlusion pressure; %EMGmax, EMG, amplitude normalized to the maximal EMG, amplitude observed during MVC, trials. Values are presented as mean ± SD. *p < 0.05, **p < 0.01, ***p < 0.001 significantly different from 0% AOP; † p < 0.05 significantly different from 60% AOP; ‡ p < 0.05 significantly different from 70% AOP; § p < 0.05 significantly different from 80% AOP. #p < 0.05 significantly different from Pre within the same condition (where applicable). F and p values represent the main effect of occlusion pressure across four levels (0%, 60%, 70%, and 80% AOP).

### 3.2 Blood lactate concentration and RPE scores

#### 3.2.1 Blood lactate (BLa)

Post-hoc tests showed that BLa at Post-0 and Post-5 was higher in 70% and 80% AOP than in 60% AOP (p < 0.05), and at Post-10 the 70% AOP condition remained higher than 80% AOP (p < 0.05). The BLa time-course is presented in Table 3.

**TABLE 3:** Blood lactate (mmol·L^-1^) dynamic changes (Mean ± SD, n = 12).

Condition	Pre-ex	Post-ex 0 min	Post-ex 5 min	Post-ex 10 min
0% AOP	3.09 ± 0.47	5.57 ± 0.41	4.01 ± 0.36	2.77 ± 0.23
60% AOP	3.08 ± 0.53	7.28 ± 0.45	5.55 ± 0.40	3.95 ± 0.28
70% AOP	3.18 ± 0.49	8.93 ± 0.70^†^	7.55 ± 0.73^†^	5.80 ± 0.54§
80% AOP	3.09 ± 0.54	8.21 ± 0.59^†^	6.85 ± 0.58^†^	5.06 ± 0.48

Pre-ex, before exercise; Post-ex, after exercise; AOP, arterial occlusion pressure. Values are presented as mean ± SD. *p < 0.05, **p < 0.01, ***p < 0.001 vs. 0% AOP; † p < 0.05 vs. 60% AOP; ‡ p < 0.05 vs. 70% AOP; § p < 0.05 vs. 80% AOP (within the same time point unless otherwise specified). #p < 0.05 vs. Pre-ex within the same pressure condition.

#### 3.2.2 Rating of perceived exertion (RPE)

There was a significant main effect of pressure on RPE scores (F (2.12, 23.33) = 34.32, p < 0.001, ηp^2^ = 0.76). Perceptual outcomes (RPE) are reported in Table 4.

**TABLE 4:** RPE scores (Borg 6–20 scale) post-exercise (Greenhouse–Geisser corrected) (Mean ± SD, n = 12).

Condition	0% AOP	60% AOP	70% AOP	80% AOP
RPE score	12.29 ± 1.14	14.08 ± 1.00*	14.38 ± 1.49*	16.33 ± 1.35*‡

RPE, rating of perceived exertion. Values are presented as mean ± SD. *p < 0.05, **p < 0.01, ***p < 0.001 vs. 0% AOP; † p < 0.05 vs. 60% AOP; ‡ p < 0.05 vs. 70% AOP; § p < 0.05 vs. 80% AOP (within the same time point). #p < 0.05 vs. Pre within the same condition (where applicable).

### 3.3 Acute changes in blood markers (IL-6, TST)

Summary results for serum biomarkers are presented in Table 5 (descriptive statistics and RM-ANOVA p-values for Time, Pressure, and Time × Pressure).

**TABLE 5:** Descriptive statistics for IL-6 and TST (mean ± SD) with RM-ANOVA p-values for Time, Pressure, and Time × Pressure.

Marker	0% AOP	60% AOP	70% AOP	80% AOP	p (Time)	p (Pressure)	p (Time × Pressure)
IL-6 (pg/mL) – Pre	1.21 ± 0.20	1.11 ± 0.20	1.12 ± 0.17	0.94 ± 0.13	<0.001	0.27	<0.001
IL-6 (pg/mL) – Post	2.51 ± 0.50	3.40 ± 0.39	3.79 ± 0.64	4.47 ± 0.63^*^	<0.001	0.27	<0.001
TST (ng/dL) – Pre	510.0 ± 22.6	511.4 ± 25.5	492.6 ± 46.7	493.5 ± 20.0	<0.001	0.957	0.009
TST (ng/dL) – Post	498.2 ± 36.4	570.2 ± 46.2^#^	588.6 ± 49.7^#^	603.8 ± 47.0^#^	<0.001	0.957	0.009

Values are presented as mean ± SD. P-values are from two-way repeated-measures ANOVA (Time, Pressure, and Time × Pressure). *p < 0.05 vs. 0% AOP, within the same time point; #p < 0.05 vs. Pre within the same pressure condition. Only *post hoc* comparisons explicitly reported in the Results are annotated with symbols.

Interleukin-6 (IL-6). Two-way repeated-measures ANOVA revealed a significant main effect of time for IL-6 (F = 348.31, p < 0.001, ηp^2^ = 0.97), no significant main effect of pressure (F = 1.37, p = 0.270, ηp^2^ = 0.11), and a significant time × pressure interaction (F = 11.70, p < 0.001, ηp^2^ = 0.52). Post-hoc comparisons indicated that IL-6 after exercise was significantly higher in the 80% AOP condition than in the 0% AOP condition (p < 0.05), suggesting an occlusion-pressure–dependent amplification of the acute inflammatory response.

Testosterone (TST). For TST, there was a significant main effect of time (F = 54.11, p < 0.001, ηp^2^ = 0.83) and a significant time × pressure interaction (F = 4.59, p = 0.009, ηp^2^ = 0.29), whereas the main effect of pressure was not significant (F = 0.10, p = 0.957, ηp^2^ = 0.01). Post-hoc tests showed that TST increased from Pre to Post in the 60%, 70%, and 80% AOP conditions (all p < 0.05), while no significant change was observed in the 0% AOP condition (p = 0.403).

## 4 Discussion

### 4.1 Main findings

The present findings demonstrate that increasing arterial occlusion pressure (AOP) during low-load isokinetic exercise augments neuromuscular activation and metabolic stress up to a certain threshold. Specifically, compared with 60% AOP, both 70% and 80% AOP elicited greater %EMGmax and blood lactate responses. However, increasing pressure from 70% to 80% AOP did not confer additional neuromuscular or metabolic benefits within the present task configuration, while perceptual strain increased markedly at 80% AOP. This pattern suggests diminishing returns beyond ∼70% AOP rather than a loss of efficacy at higher pressures. Thus, 70% AOP appears to provide a similar physiological stimulus (i.e., no additional gains relative to 80% AOP) with a lower perceptual cost under the specific velocity, load, and time frame examined in this study.

The results of this study elucidate how acute physiological responses during low-load resistance exercise are modulated by the magnitude of arterial occlusion. Overall, compared to 60% AOP, both 70% and 80% AOP conditions induced significantly higher muscle activation (%EMGmax) and blood lactate (BLa) levels. However, differences between 70% and 80% were small for %EMGmax and early lactate accumulation, although 70% remained higher than 80% at Post-10, suggesting a diminishing marginal return in neuromuscular and metabolic responses once pressure reaches a specific threshold. Conversely, the Rating of Perceived Exertion (RPE) at 80% AOP was significantly higher than at 70% AOP, indicating that extreme occlusion pressures substantially elevate the perceptual burden. This aligns with the current understanding that higher pressures in BFRT may disproportionately reduce exercise tolerability without providing additional physiological benefits (Patterson et al., 2019). In summary, while AOP≥70% provides a robust physiological stimulus, increasing pressure from 70% to 80% yields no further metabolic or electromyographic gains but significantly elevates subjective fatigue, suggesting that 70% AOP serves as a superior “stimulus-tolerance” equilibrium point for healthy adults. Notably, this pattern is consistent with recent squat-based findings using 0%–80% AOP (Zhu et al., 2025), while extending the evidence to an open-chain isokinetic dynamometry model.

### 4.2 Mechanisms of %EMGmax enhancement: hypoxia, metabolic stress, and recruitment compensation

The observed increases in %EMGmax with higher AOP should be interpreted cautiously. Surface EMG amplitude reflects the summed electrical activity of active motor units and does not directly quantify neural drive or motor unit firing rates. Therefore, elevated %EMGmax under BFRT conditions likely represents a composite effect of altered recruitment strategies, fatigue-related compensation, and peripheral signal amplification. Importantly, the apparent plateau between 70% and 80% AOP does not indicate a physiological ceiling of neuromuscular activation, but rather reflects the responses within the present isokinetic task constraints.

Consistent with previous evidence, we observed significantly higher %EMGmax under elevated AOP conditions (especially≥70%), reinforcing the efficacy of BFRT in enhancing muscle activation during low-load tasks (Scott and Loenneke, 2022). Mechanistically, BFRT induces local hypoxia and the accumulation of metabolic by-products (e.g., H+, lactate, inorganic phosphate), which collectively augment metabolic stress (Lixandrao and Ugrinowitsch, 2018). Under such conditions, low-threshold Type I muscle fibers fatigue prematurely due to inadequate oxygen supply, necessitating the compensatory recruitment of high-threshold Type II muscle fibers to maintain the target torque output (Loenneke et al., 2014). Furthermore, the accumulation of metabolites stimulates Group III/IV afferent fibers, which enhances central drive and further facilitates high-threshold motor unit recruitment (Scott and Loenneke, 2022). Our findings suggests that 70% AOP may reach the necessary threshold to trigger near-maximal recruitment compensation within the present task constraints in an isokinetic model. These mechanistic pathways are broadly consistent with prior syntheses and experimental work in BFRT (Slysz et al., 2016; Centner et al., 2019; Patterson et al., 2019). However, because within-session set-by-set EMG trajectories were not quantified in the present dataset, we cannot determine whether activation plateaued early versus increased progressively across sets; future studies should report set-level activation patterns to strengthen dose–response interpretations.

### 4.3 Metabolic response: the “plateau trend” in lactate accumulation

Metabolic stress is a hallmark of BFRT, characterized by restricted venous return that shifts energy metabolism toward anaerobic glycolysis (Patterson et al., 2019). In this study, lactate responses at 70% and 80% AOP were significantly higher than those at lower pressures, confirming that these levels provide a potent metabolic stimulus. Interestingly, the lack of a significant difference between 70% and 80% AOP suggests a “plateau trend” in metabolic response: once a sufficient degree of blood flow restriction is achieved, further increases in pressure do not linearly increase anaerobic glycolysis contribution (Patterson et al., 2019). Notably, at Post-10 min, 70% AOP maintained significantly higher BLa than 80% AOP, which may be attributed to complex microvascular dynamics and altered metabolite washout rates under varying high-pressure gradients. The relatively elevated baseline blood lactate levels may reflect dietary or recovery-related factors prior to testing; however, all conditions were compared within subjects under identical procedures, thereby preserving internal validity.

### 4.4 Perceptual burden: 80% AOP as a tolerability bottleneck

RPE increased significantly with AOP, with 80% AOP being significantly higher than both 60% and 70% AOP. RPE may also have been influenced by cuff-related discomfort/pain, particularly at higher AOP, because participants were not asked to differentiate exertion from pain-related sensations. This sharp increase in perceived exertion likely stems from the intense mechanical compression of the cuff and the amplified feedback from Group III/IV afferents sensitive to severe ischemia and metabolic acidification (Mouser and Mattocks, 2017). Given that long-term adherence to BFRT is highly dependent on subjective tolerability, the high perceptual burden associated with 80% AOP (RPE >16) may hinder protocol feasibility and sustainability (Patterson et al., 2019). Thus, 70% AOP appears to be a more viable prescription for maximizing stimuli while maintaining acceptable tolerance.

### 4.5 Blood markers: sensitivity of IL-6 and TST to pressure gradients

The significant Time × Pressure interactions observed for IL-6 and TST suggest that higher occlusion pressures amplify acute systemic responses. The pronounced increase in IL-6 at 80% AOP indicates a greater acute inflammatory or myokine response, potentially linked to a larger metabolic disturbance (Moriggi et al., 2021). Regarding TST, the significant elevation across all BFR conditions (60%–80% AOP) relative to the control condition suggests that the restricted blood flow environment is a primary driver of acute anabolic hormonal responses, likely mediated by lactate-induced signaling (Geng et al., 2021). Given the sensitivity of testosterone to circadian variation, standardized testing times across visits are important when interpreting these acute endocrine responses.

### 4.6 Practical implications and limitations

Several limitations should be acknowledged. First, muscle oxygenation was not directly assessed (e.g., via near-infrared spectroscopy), which limits mechanistic inference regarding local ischemia-hypoxia dynamics across pressure levels. Second, participants were not asked to separately rate cuff-related pain/discomfort; therefore, the extent to which occlusion discomfort contributed to RPE cannot be quantified. Third, EMG amplitude was normalized to EMGmax derived from MVC trials; we did not quantify between-visit reliability (e.g., ICC) of MVCMAX/EMGmax, and variability in maximal effort could have influenced normalized EMG outcomes. Fourth, venous blood was sampled at a fixed post-exercise time point (5 min); however, the full time course of IL-6 and testosterone responses was not characterized, and peak responses may occur at different time windows. Fifth, the absence of formal equivalence testing means that non-significant differences between 70% and 80% AOP should not be interpreted as statistical equivalence. Finally, arterial occlusion pressure is posture-dependent; because AOP was determined under standardized measurement conditions, the effective occlusion during the seated isokinetic task may have differed slightly due to hydrostatic effects. These limitations should be considered when generalizing the findings.

## 5 Conclusion

In conclusion, for acute low-load isokinetic BFRT in healthy young men, 70% AOP represents a highly practical and effective pressure prescription. Compared to lower pressures, 70% AOP induces significantly greater neuromuscular activation and metabolic stress. Compared to 80% AOP, it offers a similar physiological stimulus (without additional gains relative to 80% AOP) but with a significantly lower perceptual burden (RPE). Therefore, 70% AOP should be considered the preferred starting point for practitioners aiming to balance stimulatory efficacy with exercise tolerability.

## Data Availability

The original contributions presented in the study are included in the article/Supplementary Material, further inquiries can be directed to the corresponding authors.
