# Supplementary material for: Acute neuromuscular, metabolic, and perceptual responses to low-load isokinetic exercise under varying percentages of arterial occlusion pressure
Source: Front Physiol. 2026 Mar 4;17:1785040. doi: 10.3389/fphys.2026.1785040 (PMC12995674; doi:10.3389/fphys.2026.1785040)
Supplement: Supplementary file 1 [file Table1.docx]

**Supplementary Table S1. MVCMAX and EMGmax reference values used for EMG normalization**

Values are presented as mean ± SD (n = 12). MVCMAX was defined as the highest peak torque across MVC trials for each action at each visit (i.e., within each AOP condition), and EMGmax was defined as the peak RMS EMG (50-ms moving window) during those MVC trials (three trials; maximum retained). EMG normalization in the main analyses used visit-specific EMGmax values; the summary values reported here are participant-level MVCMAX and EMGmax measures averaged across visits for descriptive reporting.

**Table S1a. MVCMAX (peak torque) during MVC**

| Outcome | Knee extension (Nm), mean ± SD | Knee flexion (Nm), mean ± SD |
| --- | --- | --- |
| MVCMAX | 247.0 ± 25.5 | 150.1 ± 26.1 |

**Table S1b. EMGmax (maximal RMS EMG during MVC) by muscle**

| Muscle | EMGmax (µV RMS), mean ± SD |
| --- | --- |
| RF | 531.0 ± 98.7 |
| VL | 561.7 ± 133.7 |
| VM | 466.1 ± 111.4 |
| BF | 382.8 ± 98.7 |
| ST | 325.0 ± 76.7 |
| GM | 273.7 ± 66.4 |

Abbreviations: MVCMAX, peak torque during maximal voluntary contraction; EMGmax, maximal RMS EMG during MVC; RF, rectus femoris; VL, vastus lateralis; VM, vastus medialis; BF, biceps femoris; ST, semitendinosus; GM, gluteus maximus.
